# Immunometabolic crosstalk between tumor- associated macrophages and ferroptotic cancer cells: mechanisms, regulation, and therapeutic applications

**DOI:** 10.3389/fimmu.2025.1628142

**Published:** 2026-01-14

**Authors:** Zixing Qian, Zhuo Zhang, Wei Bai, Jiaxuan Li, Xianjun Rao, Guodong Huang, Jiabao Liu, Wei Wei

**Affiliations:** 1 Hubei University of Chinese Medicine, College of Traditional Chinese Medicine, Wuhan, Hubei, China; 2 Clinical College, Beijing University of Chinese Medicine, Beijing, China; 3 Wangjing Hospital, China Academy of Chinese Medical Sciences, Beijing, China

**Keywords:** cancer therapy, ferroptosis, immunometabolism, iron homeostasis, redox signaling, tumor-associated macrophages

## Abstract

Tumor-associated macrophages (TAMs) are central regulators of the metabolic and immunological landscape of solid tumors and are increasingly recognized as key determinants of cancer-cell susceptibility to ferroptosis. Ferroptosis, an iron-dependent form of regulated cell death characterized by lipid peroxidation, is tightly shaped by metabolic cues within the tumor microenvironment (TME). TAMs, through their remarkable metabolic plasticity, modulate iron flux, redox balance, polyunsaturated fatty-acid (PUFA) availability, and glutathione-dependent antioxidant pathways, each of which directly influences ferroptotic vulnerability in neighboring tumor cells. In this review, we synthesize current evidence linking TAM polarization states to the regulation of ferroptosis-related processes, including lipid remodeling, cystokine metabolism, reactive oxygen species (ROS) buffering, and immunometabolic signaling. We further discuss how TAM-derived cytokines, lipid mediators, and iron-handling proteins orchestrate a microenvironment that either promotes or restrains ferroptotic cell death. Finally, we highlight emerging therapeutic strategies aimed at rewiring TAM metabolism or exploiting ferroptosis to overcome immune suppression and therapy resistance. By integrating immunological and metabolic dimensions, this review provides a framework for understanding TAM-ferroptosis crosstalk and its implications for precision immunotherapy in cancer.

## 1 Introduction

Tumor-associated macrophages (TAMs), a dominant immune cell population in the tumor microenvironment (TME), significantly influence cancer progression (1, 2). While typically associated with poor clinical outcomes due to their potent immunosuppressive and wound-healing properties, TAMs promote metastasis, pro-oncogenic signaling, angiogenesis, and ECM remodeling (3, 4). Intriguingly, TAM function is context- and treatment-dependent, exhibiting anti-tumorigenic effects in certain situations, either directly or by enhancing CD8+ T cell cytotoxicity. This functional plasticity and heterogeneous nature across tumors, patients, and species complicate their impact on tumor biology, particularly in human malignancies, which present a more complex picture than the predominantly pro-tumorigenic TAM activity observed in rodent models (5, 6). Some high-burden malignancies, such as colorectal (CRC), head and neck (HNC), lung (lung cancer), prostate (prostate cancer), and esophageal (gastric cancer) cancers, have shown mixed or positive prognostic implications for TAMs, rather than only negative ones (7). Given the high genetic variability among human populations, the remarkable adaptability and plasticity of TAMs are likely responsible for their varied clinical effects.

**Figure 1:**
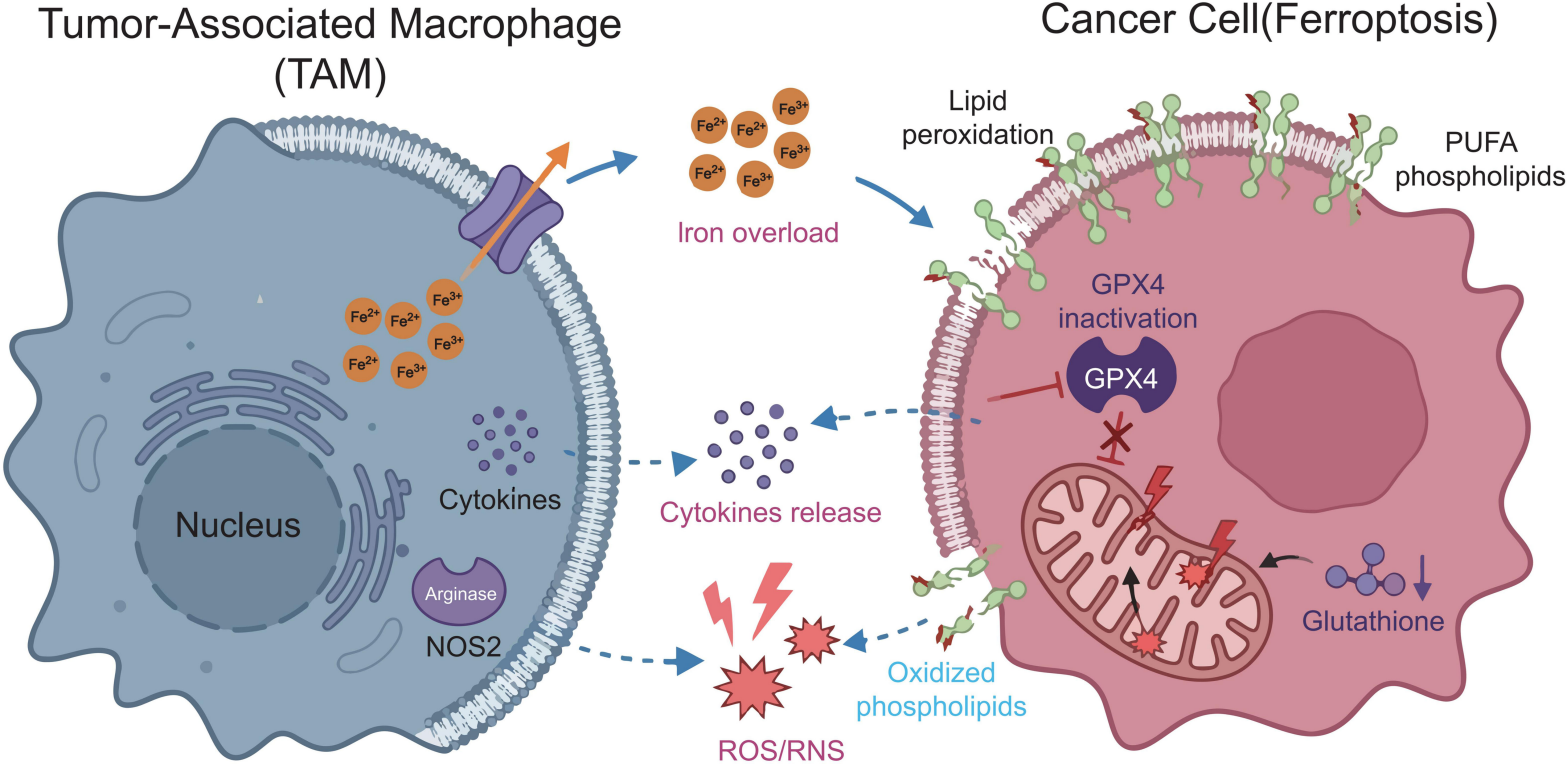
Metabolic immune crosstalk between TAMs and cancer-cell ferroptosis. It emphasizes crosstalk between TAMs and cancer cells undergoing ferroptosis, depicting how TAM metabolic activities affect cancer-cell ferroptosis, and how cancer-cell ferroptosis modulates TAM immune functions.

Ferroptosis represents a metabolically driven form of regulated cell death that is fundamentally distinct from apoptosis, necroptosis, and pyroptosis (8). Unlike apoptosis, which is characterized by caspase activation and DNA fragmentation, or necroptosis and pyroptosis, which are mediated by RIPK1/RIPK3–MLKL or gasdermin pore formation, respectively, ferroptosis is uniquely governed by iron-dependent lipid peroxidation and redox imbalance. Its execution hinges on the accumulation of oxidized polyunsaturated fatty acid containing phospholipids (PUFA-PLs), depletion of intracellular glutathione (GSH), and inactivation of glutathione peroxidase 4 (GPX4). These metabolic vulnerabilities arise from disruptions in cytokine import, glutathione synthesis, CoQ10 regeneration, and the signaling of ferroptosis-suppressor protein 1 (FSP1). In contrast to other RCD pathways, ferroptosis critically relies on iron trafficking, ferritinophagy, and expansion of the labile iron pool, making it uniquely sensitive to metabolic cues within the tumor microenvironment. Tumor-associated macrophages (TAMs), through their specialized iron handling, lipid remodeling programs, and redox-modifying functions, are positioned as central regulators of ferroptotic susceptibility in neighboring cancer cells (9). Understanding these metabolic distinctions is crucial for comprehending why TAMs exert profound control over ferroptosis and how their reprogrammed immunometabolic states can influence tumor progression and therapeutic response, as shown in **Figure 1**.

Ferroptosis, a recently identified form of programmed cell death, is characterized by elevated levels of reactive oxygen species (ROS) and iron-dependent lipid peroxidation (10). The metabolic pathways regulating iron, glucose, and amino acids confer distinct biochemical and genetic traits on ferroptosis, setting it apart from apoptosis, necroptosis, and pyroptosis (11). Ferroptosis is predicated on dysregulated iron homeostasis as its underlying mechanism. A reduction of Fe³^+^ to Fe²^+^ occurs after cellular iron uptake through transferrin receptor 1 (TFR1). The labile iron pool is a site of excess iron accumulation, while ferritin complexes composed of heavy (FTH) and light chains (FTL) represent another location for iron storage. Intracellular iron accumulation is significantly facilitated by diminished expression of ferroportin (FPN), the sole confirmed iron exporter (12). This then induces iron ion Fenton reactions, generating reactive oxygen species (ROS) that damage cells and activate lipoxygenases (LOXs), resulting in lethal lipid peroxidation. The downregulation of the cystine/glutamate transporter (system Xc^-^) constitutes the initial ferroptotic signal. This diminishes cystine absorption and glutathione production, thereby accelerating the formation of lipid-reactive oxygen species (13). Two essential regulatory genes, SLC7A11 and GPX4, play a crucial role in ferroptotic processes. The role of ferroptosis in numerous clinical diseases has been thoroughly studied. This encompasses inflammatory disorders (including acute kidney injury, neuroinflammation, and atherosclerosis) (14, 15), cancers (such as laryngeal, hepatocellular, breast, pancreatic, lung, and brain malignancies) (16, 17), infectious diseases (like tuberculosis and pyemia) (18, 19), and neurodegenerative and systemic disorders (including Alzheimer’s disease and leukemia) (20, 21). This list is not comprehensive. This research highlights the potential of pharmacological modulation of ferroptosis, either by inhibition or stimulation, as a therapeutic approach for illnesses associated with dysregulated lipid peroxidation (22).

This review offers a comprehensive and critical examination of recent data on the bidirectional relationships and molecular pathways linking macrophage biology to ferroptotic cell death processes in various clinical settings. We consolidate existing findings on the therapeutic potential of targeting the macrophage-ferroptosis axis, with a specific focus on oncological applications. Additionally, we recognize substantial knowledge deficiencies and unanswered questions in this domain, which we hope will encourage future research avenues. By emphasizing these research priorities, we aim to stimulate scientific progress that could lead to innovative intervention strategies for ferroptosis-related disorders, particularly in malignancies where macrophage function and iron metabolism present promising therapeutic targets.

## 2 Tumor-associated macrophages: an in-depth analysis

The traditional belief that tumor-associated macrophages (TAMs) solely originate from bone marrow monocytic progenitors has evolved substantially in recent years (23). Current evidence suggests that, although this mechanism is present in various animal models, tissue-resident macrophages, derived from embryonic precursors, significantly augment the tumor-associated macrophage population under homeostatic conditions, alongside the bone marrow-derived subset (24). Upon recruitment to neoplastic sites or differentiation from resident populations within the tissue of origin, these macrophages undergo phenotypic reprogramming specific to the tumor microenvironment, resulting in discrete tumor-associated macrophage subpopulations. As the primary infiltrating immune cell type within tumors, TAMs have gained substantial scientific attention due to their crucial involvement in neoplastic growth, metastatic dissemination, and treatment resistance mechanisms (25). The subsequent subsections clarify the essential elements of TAMs, focusing specifically on ontological frameworks and terminological standards commonly used in cancer research.

### 2.1 Diversity and classifications of TAM

**Figure 2:**
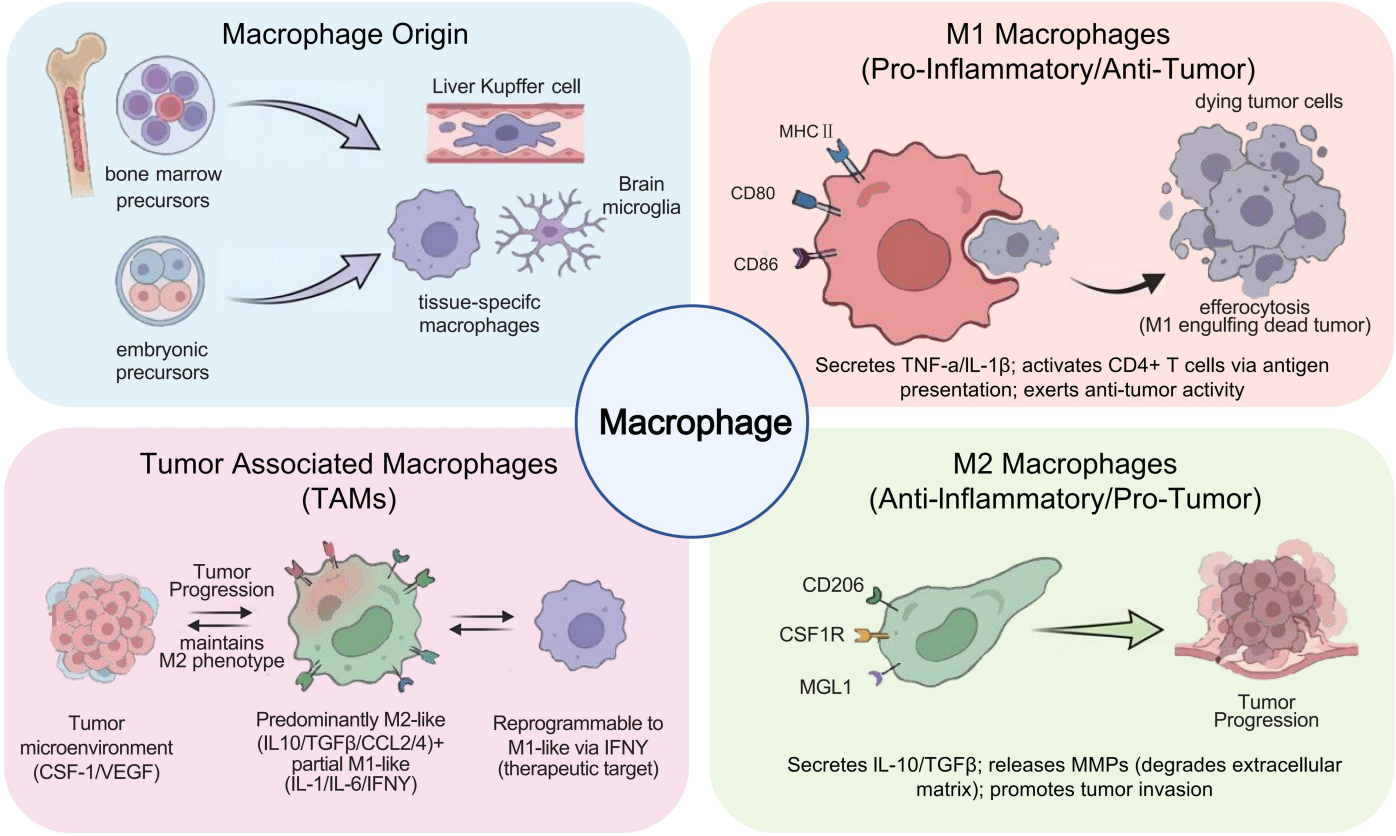
Schematic overview of macrophage developmental origin, functional polarization subtypes, and their regulatory roles in tumor microenvironment.

The diverse origins and functional variability of TAMs (**Figure 2**) pose considerable difficulties for their ontological classification (26). Historically, tumor-associated macrophages were defined as immune cells capable of phagocytosing cancer cells, resulting in lethal consequences, and were initially categorized as anti-tumorigenic entities (27). Recent studies, however, have demonstrated their primary tumor-promoting role (28). The notable plasticity and diversity of tumor-associated macrophages, due to intra-tumoral heterogeneity and inter-patient variability, complicates the identification of consensus biomarkers or ontological systems that can differentiate between tumor-promoting and anti-tumor TAM phenotypes. TAM features can be dynamically changed during tumor growth or in response to therapeutic interventions, including targeted therapies and conventional radiotherapy or chemotherapy regimens (29). The biological complexities have made TAM definitions and ontologies dependent on the dimensionality of the analytical methods available for their development. Initially, flow cytometry enabled the preliminary classification of tumor-associated macrophage subsets (30). However, multidimensional techniques have refined or complicated these classifications, including single-cell omics technologies (31). Various TAM classification schemes exist, each relying on health versus illness states, particular clinical situations, tissue type specificity, or functional activity patterns.

### 2.2 Macrophage polarization states and their distinctive properties

Macrophages exhibit remarkable heterogeneity and adaptability, rapidly responding to environmental cues by polarizing into functionally distinct phenotypes. Under stimulation by lipopolysaccharide (LPS) and interferon-γ (IFN-γ), macrophages adopt the M1 phenotype (32). These classically activated M1 macrophages produce pro-inflammatory mediators, including IFN-γ, tumor necrosis factor-α (TNF-α), and inducible nitric oxide synthase (iNOS) (33). Metabolically, M1 macrophages exhibit enhanced glycolytic activity, elevated ferritin expression, reduced membrane iron transporter levels, increased glutathione content, upregulated cyclooxygenase-2 (COX2) with concurrent downregulation of cyclooxygenase-1 (COX1), and heightened iNOS2 activity alongside diminished arginase-1 (Arg1) function (34). Conversely, cytokines such as IL-4, IL-13, and macrophage colony-stimulating factor (M-CSF) drive polarization toward the M2 phenotype, which secretes anti-inflammatory factors including IL-4, IL-10, and IL-13 (35). M2 macrophages characteristically express elevated levels of mannose receptor (CD206), resistin-like molecule α, Arg1, and chitinase. The production of IL-10 and IL-1 receptor antagonists generates various anti-inflammatory cytokines, which are essential for tissue repair and remodeling following pathogen-induced damage (36, 37).

## 3 Macrophage metabolic states and tumor function

Macrophage polarization is tightly governed by distinct metabolic programs that determine their effector functions within the tumor microenvironment (38). Classically activated M1 macrophages adopt a glycolytic metabolic profile characterized by rapid glucose uptake, enhanced lactate production, and interruption of the tricarboxylic acid (TCA) cycle at the citrate and succinate nodes. This metabolic rewiring supports nitric oxide (NO) synthesis via inducible nitric oxide synthase (iNOS), drives the accumulation of reactive oxygen species (ROS), and fuels the production of pro-inflammatory cytokines. Elevated ACSL4 activity, increased ferritin expression, and restricted iron export further enhance the oxidative and iron-dependent milieu, enabling M1 macrophages to promote ferroptotic pressure on neighboring tumor cells (39). Conversely, alternatively activated M2 macrophages rely predominantly on oxidative phosphorylation (OXPHOS), fatty-acid β-oxidation (FAO), and an intact TCA cycle. These pathways sustain tissue-repair programs through the production of ornithine, polyamines, and anti-inflammatory cytokines such as IL-10 and TGF-β. M2 macrophages exhibit higher ferroportin levels, increased heme recycling, and robust glutathione synthesis, collectively establishing a ferroptosis-resistant environment that enables tumor progression and immune evasion. Additionally, lipid-associated TAM subsets utilize cholesterol efflux pathways, PPAR signaling, and scavenger receptor-mediated uptake to fine-tune their immunosuppressive functions within the TME (40). These divergent metabolic signatures are not merely byproducts of polarization but active determinants of macrophage behavior. By shaping redox tone, iron availability, lipid composition, and cytokine output, macrophage metabolic states critically influence ferroptotic susceptibility in adjacent cancer cells and define their overall contribution to tumor development.

### 3.1 Oncogenic functions of tumor-associated macrophages

Tumor-associated macrophages (TAMs) represent macrophages recruited and reprogrammed by the tumor microenvironment (TME), exhibiting distinct functional differences from conventional immune macrophages. M2-polarized TAMs collaborate with angiogenic factors, including epidermal growth factor (EGF) and placental-derived growth factor, within the TME. Their secretion of matrix metalloproteinases (MMPs), serine proteases, and cathepsins disrupts the endothelial basement membranes, degrades extracellular matrix components, including collagen, and facilitates the migration of tumor and stromal cells, ultimately promoting tumor angiogenesis and metastasis (41). The tyrosine-protein kinase receptor 2 (Tie2) expressed by specific TAM subsets interacts with various angiopoietins to accelerate tumor dissemination. Therapeutic targeting of Tie2 significantly reduces TAM presence in tumors, effectively controlling cancer cell proliferation and spread (8), thereby inhibiting tumor growth. TAMs regulate tumor cell proliferation through numerous growth factors and receptors, including EGF, platelet-derived growth factor, transforming growth factor-β1 (TGF-β1), hepatocyte growth factor, epidermal growth factor receptor (EGFR) ligands, and basic fibroblast growth factor (42). Additionally, TAMs indirectly modulate tumor growth through complex signaling networks affecting immune and angiogenic cells within the TME. These regulatory mechanisms encompass gene expression, protein modification, and intercellular communication, with TAMs precisely orchestrating tumor cell growth, development, invasion, and metastasis through diverse signaling pathways (43, 44).

**Figure 3:**
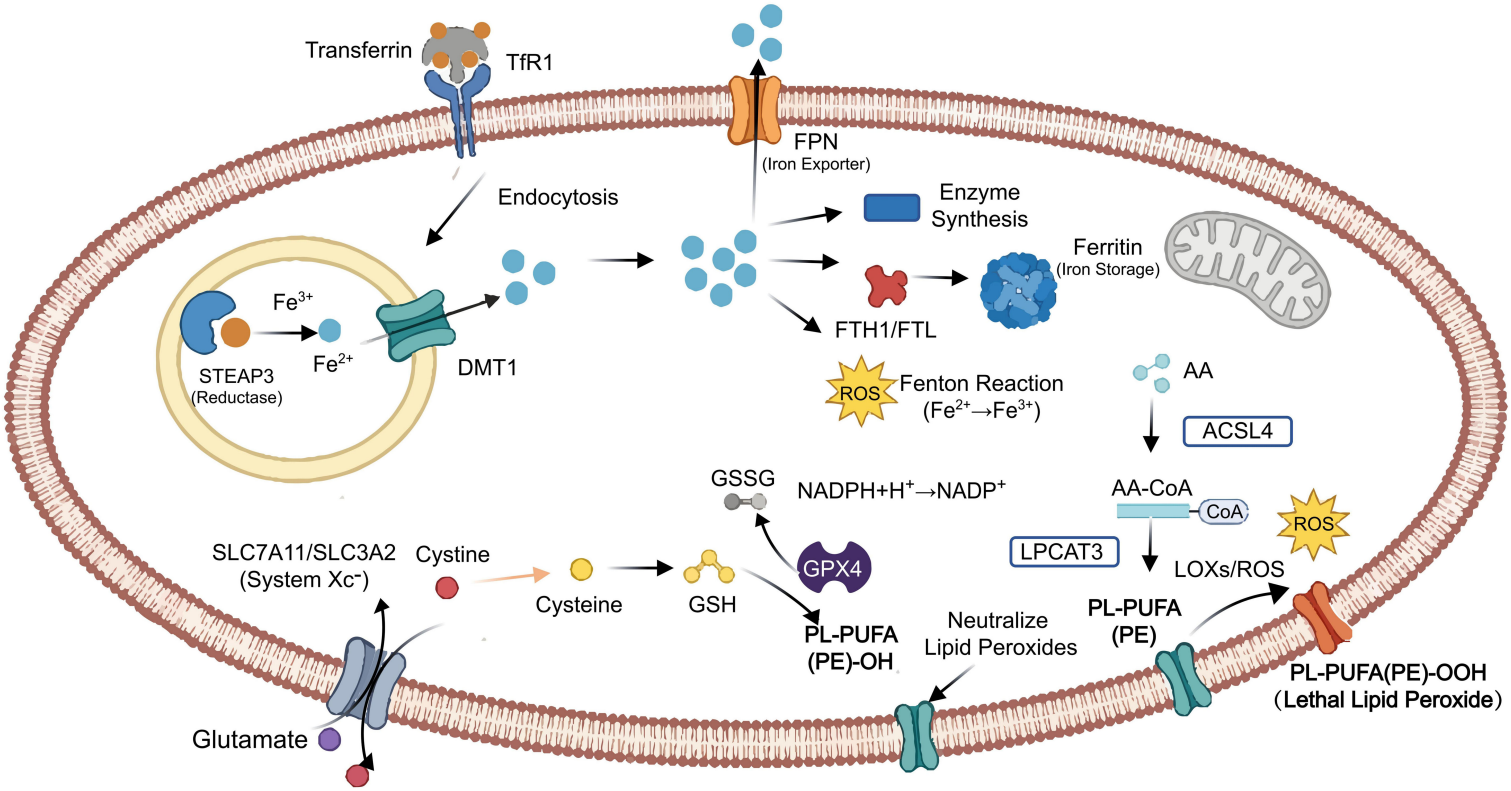
Molecular mechanism of ferroptosis. Ferroptosis involves iron import (TFR1, STEAP3, FPN), ROS generation, cystine import (SLC7A11/SLC3A2) for GSH/GPX4-mediated lipid peroxide neutralization, and PUFA processing (ACSL4, LPCAT3) into peroxidizable membrane phospholipids. Failed antioxidant defenses lead to the accumulation of PL-PUFA(PE)-OOH, triggering cell death.

Dixon defined ferroptosis as a unique type of controlled cell death in 2012, based on its iron-dependent mechanism and fundamental deviations from apoptotic pathways (45, 46). Lipid hydroperoxides accumulating gradually inside cell membranes define this process. Among other illnesses of the cardiovascular system, neurological ailments, hepatotoxicity, and renal damage, a growing body of evidence has connected ferroptotic pathways to the etiology of numerous clinical conditions (1–4). Ferroptosis’s biochemical signature involves the oxidative degradation of phospholipid membranes through both enzyme-catalyzed and spontaneous chemical processes (47, 48). Key molecular features of ferroptotic cells include abnormal iron accumulation and dysfunction of glutathione peroxidase 4 (GPX4), thereby highlighting the complex interplay of this process with cellular pathways regulating iron homeostasis, lipid metabolism, and amino acid processing (**Figure 3**).

### 3.2 Integrated immune metabolic regulation of ferroptosis

**Figure 4:**
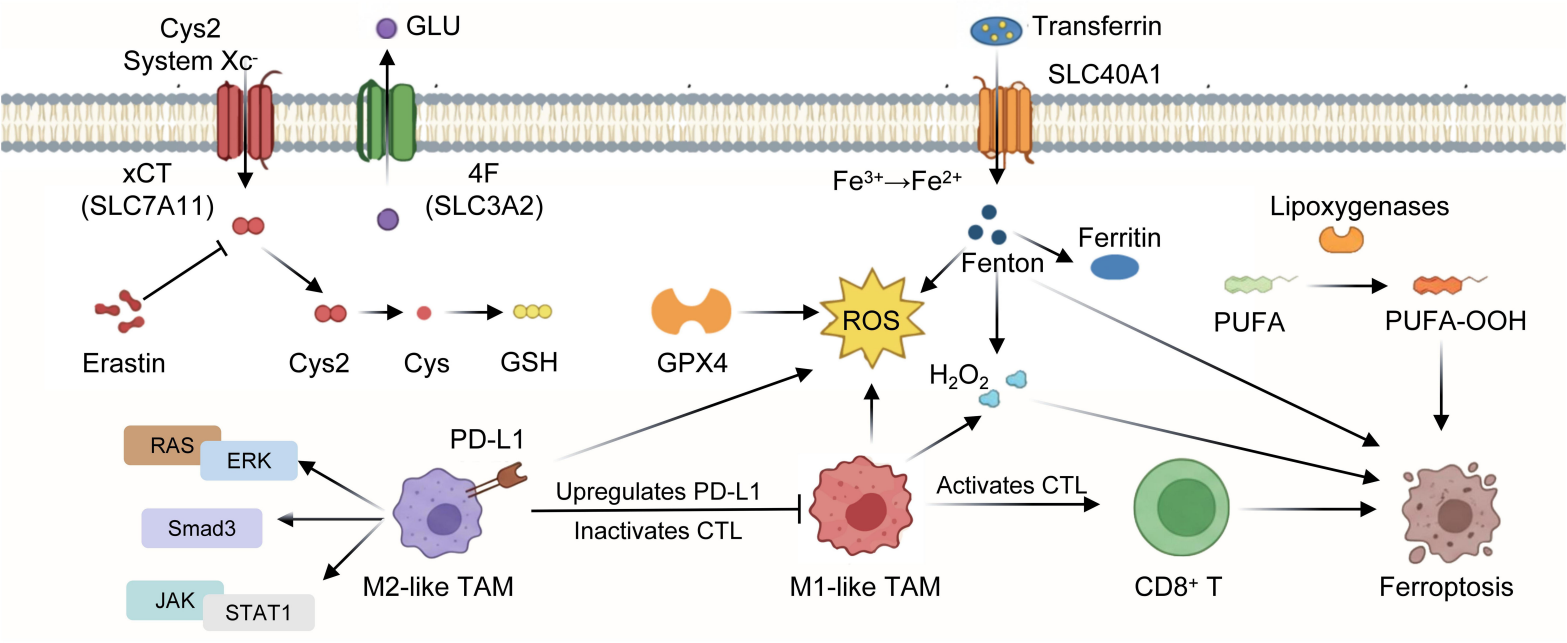
Cellular regulation of ferroptosis and TAM-mediated effects. System Xc⁻ (SLC7A11/SLC3A2) mediates cystine uptake (for glutathione [GSH] synthesis, blocked by Erastin), and GPX4 uses GSH to scavenge ROS; iron drives ROS production via the Fenton reaction, while lipoxygenases oxidize PUFA—both triggering ferroptosis. Meanwhile, M2-like TAMs upregulate PD-L1 to inactivate CTLs, whereas M1-like TAMs activate CTLs to promote ferroptosis.

Ferroptosis involves aberrant iron metabolism, resulting in lipid peroxidation and the degradation of cellular membranes. Typically, extracellular Fe³^+^ is sequestered by transferrin, then reduced to Fe²^+^ within the cell, with any surplus exported by SLC40A1 (49). Excess Fe²^+^ is usually sequestered in ferritin; when this process is compromised, Fe²^+^ activates lipid peroxidases, producing reactive oxygen species (50), which oxidize membrane polyunsaturated fatty acids (PUFAs) to PUFA-OOHs, resulting in irreversible membrane damage and cellular apoptosis (**Figure 4**). Intracellular and extracellular mechanisms govern Ferroptosis inside the tumor immune microenvironment (TIME). The primary intracellular regulator is the Xc^-^/GSH/GPX4 axis. System Xc^-^, a heterodimer composed of xCT (SLC7A11) and 4F2 (SLC3A2) (51), facilitates the import of cystine and the export of glutamate, hence supplying precursors for glutathione production. GSH mitigates reactive oxygen species and lipid hydroperoxides through the action of GPX4. Ferroptosis inducers such as erastin obstruct this route by inhibiting SLC7A11, while concurrently activating mitochondrial VDACs, upregulating ACSL4, and indirectly stimulating p53. Recently discovered GSH-independent compounds, such as FSP1, also modulate tumor ferroptosis (52).

CD8^+^ T cells predominantly trigger ferroptosis in malignancies through extracellular mechanisms. They demonstrated that T cell-derived IFN-γ activates the JAK/STAT1 pathway, resulting in the formation of STAT1 homodimers that inhibit SLC7A11 transcription (53, 54). IFN-γ also elevates mitochondrial reactive oxygen species, presumably via the STAT1/NF-κB/NOS2 pathway. Furthermore, IFN-γ activates ACSL4, enabling the integration of arachidonic acid into phospholipids and thereby enhancing oxidative susceptibility (55, 56). Although IFN-γ augments the anti-tumor efficacy of CD8^+^ T cells, the tumor immune microenvironment (TIME) considerably affects this mechanism, possibly restricting ferroptosis in immunologically inert malignancies (57, 58). Identifying essential TIME regulators is vital for advancing effective ferroptosis-based cancer treatments.

## 4 Ferroptosis and cell death in tumor immunology

Ferroptosis represents a unique form of regulated cell death characterized by excessive accumulation of lipid peroxides and reactive oxygen species (ROS). Beyond its involvement in disease-related signaling pathways, ferroptosis is intricately regulated by the metabolism of iron, carbohydrates, and amino acids. Dysregulated iron homeostasis constitutes the fundamental basis for ferroptosis initiation (59). The central mechanism driving ferroptosis involves the pathological accumulation of toxic polyunsaturated fatty acid-phospholipid-hydroperoxides (PUFA-PLOOH), resulting from enhanced synthesis and impaired degradation processes.

### 4.1 Molecular pathways governing ferroptosis cell death

Iron is a critical driver of lipid peroxidation and subsequent ferroptosis within cellular environments (60, 61). Research demonstrates that various iron metabolism-related genes regulate ferroptosis processes, including transferrin, nitrogen fixation system 1 (NFS1), iron-responsive element-binding protein 2 (IREB2), nuclear receptor co-activator 4 (NCOA4), solute carrier family 7 member 11 (SLC7A11), and glutathione peroxidase 4 (GPX4)—each representing key genetic determinants of ferroptosis susceptibility (62, 63).

Diminished activity of the cystine/glutamate antiporter (system Xc-) serves as the initial trigger for ferroptosis (64, 65). When the system Xc-function becomes compromised, cellular cystine uptake decreases, leading to reduced glutathione (GSH) synthesis and consequent elevation of lipid ROS levels, ultimately culminating in ferroptosis cell death (66).

Ferroptosis substrates primarily include polyunsaturated fatty acids (PUFAs, characterized by their abundance of double bonds), free polyunsaturated fatty acids such as oxidized arachidonic acid, and adrenal hormones. These fatty acids induce ferroptosis through esterification and incorporation into cellular membrane phospholipids (67). Intracellularly, ROS facilitate the integration of oxidized free polyunsaturated fatty acid products into biological membranes, including plasma and mitochondrial membranes, as shown in **Table 1**. This integration compromises membrane function, reducing biological membrane fluidity and leading to impaired cellular energy production, disruption of biological membrane integrity, and restricted material exchange, collectively disrupting normal cellular physiology.

**Table 1:** Key molecular players in ferroptosis and TAM interactions.

Molecular player	Function in ferroptosis	Role in TAM biology	Therapeutic potential	Clinical evidence
GPX4	Reduces lipid peroxides; primary ferroptosis suppressor	Regulates M1/M2 polarization via ROS control	Target for ferroptosis induction	Phase I/II trials ongoing
System Xc- (SLC7A11)	Cystine uptake for GSH synthesis	Modulates inflammatory responses	Erastin, SAS inhibition	FDA-approved SAS
ACSL4	Incorporates PUFAs into phospholipids	Enhanced by IFN-γ from activated macrophages	Biomarker for ferroptosis sensitivity	Prognostic marker studies
TFR1 (CD71)	Iron uptake via transferrin	Higher in M1 TAMs; iron sequestration	Iron-based nanoparticle targeting	Ferumoxytol FDA-approved
Ferroportin (FPN)	Iron export	Higher in M2 TAMs; promotes tumor growth	Regulate iron availability	Preclinical studies
HMGB1	DAMP release during ferroptosis	Macrophage recruitment and activation	Anti-HMGB1 antibodies	Clinical trials for sepsis
SAPE-OOH	“Eat-me” signal on ferroptotic cells	Enhances phagocytosis via TLR2	Improve immunotherapy efficacy	Research phase
NRF2	Antioxidant response activation	Ferroptosis resistance in TAMs	Combination with ferroptosis inducers	Clinical trials

### 4.2 Immunometabolic crosstalk between macrophages and ferroptotic cancer cells

Macrophages provide essential material requisites for ferroptosis through their ability to uptake and store iron ions. When macrophages encounter specific stimuli, they phagocytose ferroptotic pancreatic cancer cells harboring KRAS-G12D mutations. This interaction promotes M2 macrophage polarization, enhancing pro-tumorigenic phenotypes (68). Infection, injury, and inflammation stimulate macrophage iron uptake and storage, thereby facilitating the induction of ferroptosis. M1-polarized macrophages secrete ferroptosis-inducing factors that act on neighboring cells and efficiently eliminate post-ferroptotic necrotic cells through phagocytosis. During this process, macrophages perform crucial clearance functions that maintain tissue and organ homeostasis (69). Conversely, M2-polarized macrophages secrete cytokines such as IL-10 and TGF-β, which activate anti-ferroptotic signaling cascades within tumor cells. These cascades regulate antioxidant enzymes, such as GPX4, inhibit lipid peroxidation reactions, and suppress tumor cell ferroptosis.

### 4.3 Macrophage-ferroptosis immunometabolism

Tumor-associated macrophages (TAMs) and ferroptotic cancer cells engage in a highly integrated, bidirectional metabolic dialogue that shapes immune outcomes and determines tumor (70). This crosstalk is governed by iron flux, lipid remodeling, amino-acid metabolism, mitochondrial redox balance, and cytokine-driven signaling loops. M1-like macrophages amplify ferroptotic pressure through ferritinophagy-mediated release of labile Fe²^+^, increased transferrin receptor (TfR1) expression, and restricted ferroportin activity, thereby enriching the tumor microenvironment (TME) with redox-active iron (71). Elevated nitric oxide (NO), reactive oxygen species (ROS), and pro-inflammatory cytokines (IL-1β, TNF-α, IFN-γ) further inhibit SLC7A11 and destabilize GPX4 activity in cancer cells. These macrophage-derived metabolic signals synergistically increase lipid peroxidation through the ACSL4-dependent synthesis of PUFA-phospholipids, pushing cancer cells beyond their antioxidant capacity and into a state of ferroptosis. By contrast, M2-like TAMs create a ferroptosis-resistant niche through high ferroportin expression, heme degradation pathways (HMOX1), the generation of antioxidant glutathione, and the secretion of IL-10 and TGF-β (72). These cues reinforce GPX4 activity, suppress lipid peroxidation, and modulate NADPH-dependent regeneration pathways, collectively shielding tumor cells from ferroptotic damage. Lipid-associated TAM subsets additionally supply oxidized cholesterol derivatives and anti-inflammatory lipid mediators that reinforce immune tolerance and inhibit ferroptosis execution.

**Figure 5:**
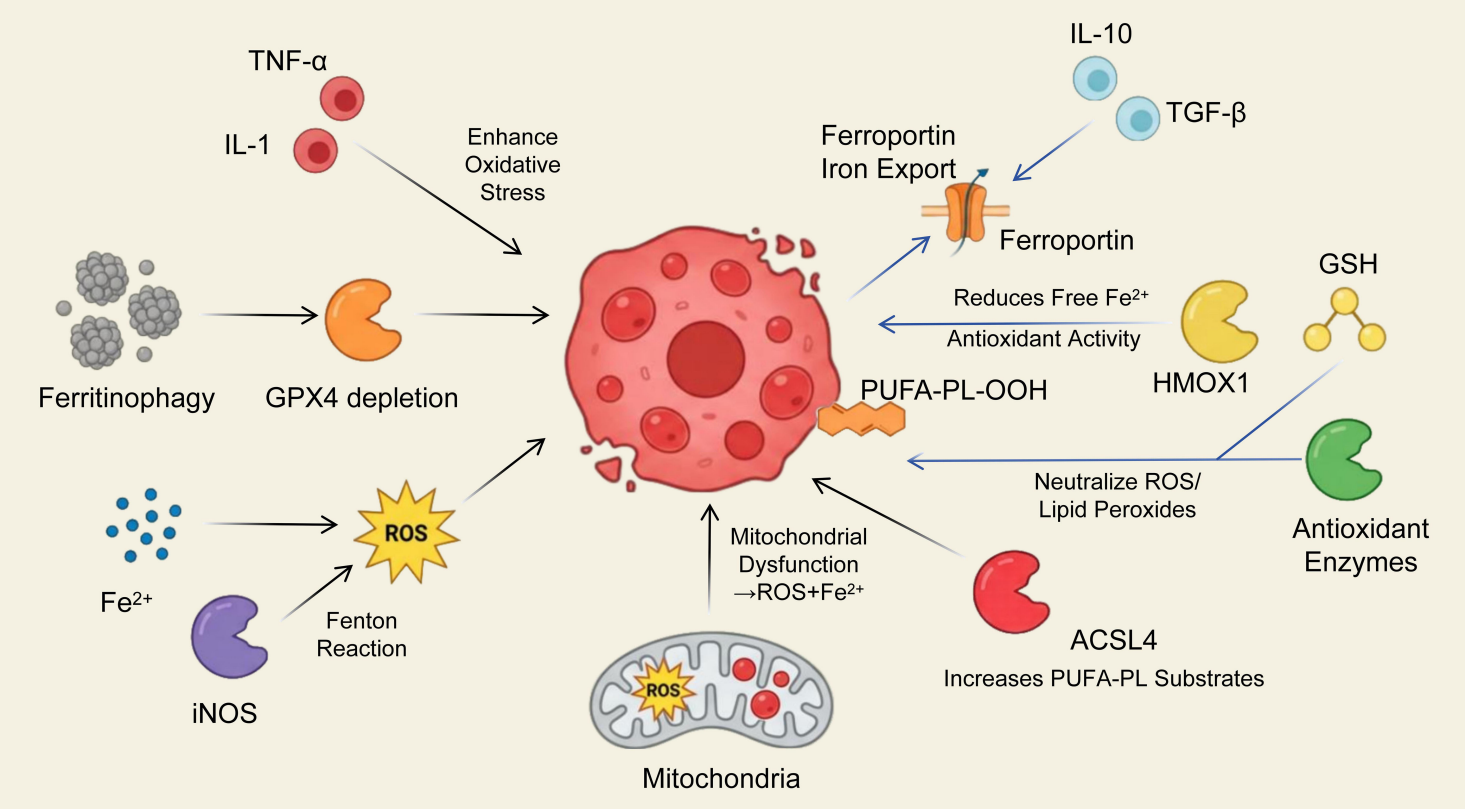
TAMs influence how immune cell metabolism affects cancer cell ferroptosis, thereby shaping the tumor microenvironment. Studying these immunometabolic links is crucial for cancer therapy, particularly the reciprocal role of TAM metabolism in cancer cell ferroptosis.

Ferroptotic cancer cells reciprocally influence macrophage states by releasing DAMPs (HMGB1), oxidized phosphatidylethanolamines (SAPE-OOH), iron-loaded vesicles, and lipid peroxidation products that serve as signals to recruit, polarize, or reprogram macrophages (73). Early ferroptotic intermediates can promote M1 activation, whereas chronic ferroptotic stress and lipid peroxides tend to skew macrophages toward immunosuppressive phenotypes, as shown in **Figure 5**. These reciprocal loops define a dynamic immunometabolic axis that determines whether ferroptosis amplifies anti-tumor immunity or reinforces tumor tolerance.

### 4.4 Direct TAM cancer cell ferroptosis interactions: a mechanistic narrative

Tumor-associated macrophages (TAMs) orchestrate ferroptotic sensitivity in cancer cells through a set of tightly interlinked metabolic and immunological interactions (74). M2-like TAMs, which dominate many solid tumors, create a ferroptosis-resistant niche by exporting iron through ferroportin, supplying cysteine precursors that support glutathione synthesis, and releasing IL-10, TGF-β, and lipid mediators that dampen oxidative stress. These signals collectively stabilize GPX4 activity and limit lipid-peroxide accumulation, thereby shielding tumor cells from ferroptotic death. In contrast, M1-like TAMs impose a ferroptosis-promoting environment through ferritin degradation (ferritinophagy), release of labile iron, production of nitric oxide and ROS, and secretion of pro-inflammatory cytokines that suppress SLC7A11 function and perturb GSH biosynthesis (75). Through ACSL4 induction and provision of oxidizable PUFA substrates, M1 TAMs directly enhance the lipid-peroxidation machinery necessary for ferroptosis execution.

Bidirectional signaling further reinforces this crosstalk: tumor-cell–derived lactate, HMGB1, prostaglandins, and oxidized lipids polarize macrophages toward either ferroptosis-promoting (M1-like) or ferroptosis-restraining (M2-like) states. Ferroptotic cancer cells also release danger-associated lipids and iron intermediates that recruit and activate macrophages, creating positive or negative regulatory loops (76). This integrated metabolic–immune dialogue determines whether ferroptosis proceeds or is suppressed within the tumor microenvironment, representing a central axis of TAM-mediated control over tumor progression.

## 5 Iron homeostasis and immune regulation in the tumor microenvironment

### 5.1 Impact of macrophage polarization states on iron metabolism

During early tumorigenesis, M1-type tumor-associated macrophages (TAMs) facilitate cellular iron uptake via transferrin receptor 1 (TfR1/CD71), while ferritin (FT) restricts iron efflux, resulting in iron sequestration. Pro-inflammatory cytokines, including IL-6, IL-1, and TNF-α, promote M1-type TAM formation, inhibit iron release into the tumor immune microenvironment (TIME), and exert anti-tumor effects (77). In circulation, ferric ions (Fe³^+^) bind to FT for transport throughout the body. Upon reaching target tissues, iron is absorbed by cells through the transferrin receptor 1 (TfR1) receptor. Intracellularly, iron undergoes reduction to its ferrous form (Fe²^+^) by the reductase six-transmembrane epithelial antigen of prostate 3 (STEAP3) before entering the cytoplasm through divalent metal transporter 1 (DMT1). TfR1 recycles to the cell surface during this process for continued iron transport. Regulating TAM iron metabolism within the TIME, specifically by promoting iron uptake and storage in M2-type macrophages while inhibiting iron release, allows iron accumulation in TAMs to enhance ROS production, increase p300/CBP acetyltransferase activity, and promote p53 acetylation. These changes drive conversion to the pro-inflammatory M1 phenotype, which exhibits anti-tumor properties (78). In contrast, M2-type macrophages demonstrate enhanced iron efflux capacity through higher ferroportin (FPN) expression and lower FT levels, inducing elevated CD91 or CD163 expression (79). CD163, a cell surface protein with high binding affinity for specific substrates, is a distinctive M2-type TAM marker. These macrophages play significant roles in the tumor microenvironment, typically promoting tumor growth and dissemination. CD163 facilitates efficient hemoglobin phagocytosis by these macrophages, enabling iron acquisition. As iron is essential for cellular proliferation, CD163 indirectly supports tumor cell growth (80).

M2-type TAMs enhance intracellular heme accumulation by phagocytosing senescent erythrocytes. Following heme internalization, heme oxygenase-1 (HMOX-1) degrades heme to produce bilirubin, carbon monoxide, and ferrous ions, which inhibit the interactions of iron-binding proteins with iron response elements in FPN mRNA, thereby upregulating FPN expression and promoting tumor growth (81). Consequently, M2-type TAMs within the TIME facilitate iron transport to the extracellular environment, suppress recruitment and cytotoxic function of tumoricidal immune cells, stimulate angiogenesis, and enhance tumor cell proliferation, invasion, and metastasis. Leveraging the relationship between macrophage polarization phenotypes and iron metabolism, therapeutic targeting of TAMs within the TME represents a promising anti-tumor strategy (2). Researcher demonstrates that tumor-delivered iron nanoparticles, when internalized by TAMs, promote their conversion to tumor-suppressive M1-type macrophages (82). The FDA-approved iron supplement ferumoxytol (an iron oxide nanoparticle) has been shown to inhibit subcutaneous adenocarcinoma development in mice by promoting TAM polarization toward the M1 phenotype and preventing hepatic tumor metastasis. Further investigation is needed to determine the optimal M1/M2 macrophage balance in relation to iron metabolism within the tumor microenvironment.

### 5.2 Macrophage-mediated regulation of tumor cell ferroptosis

Macrophages exhibit remarkable plasticity, changing their phenotype and functional spectrum in response to the microenvironment, thereby demonstrating the existence of heterogeneous subpopulations. Macrophage polarization has a critical influence on tumor development and the regulation of ferroptosis. Traditionally, activated macrophages are classified into two main types: proinflammatory M1 macrophages and anti-inflammatory M2 macrophages. However, this binary classification has faced recent criticism (83). M1 macrophages, induced by Toll-like receptor ligands (e.g., LPS) or Th1 cytokines such as TNF-α, IFN-γ, and colony-stimulating factor 2 (CSF2), are characterized by surface expression of TLR2, TLR4, CD80, and CD86 (84). With high antigen-presenting capacity, they secrete reactive oxygen species (ROS) and proinflammatory cytokines, including IL-1, IL-6, IL-12, IL-18, IL-23, and TNF-α, which modulate Th1-mediated antigen-specific inflammatory responses (85, 86). M1 macrophages enhance inducible nitric oxide synthase (NOS2 or iNOS) expression, thereby promoting NO production from L-arginine (79). Their infiltration is considered a favorable prognostic factor in tumors (87, 88). These M1-derived inflammatory mediators have a significant impact on ferroptosis pathways. Notably, macrophage-derived TNF-α plays a role in modulating ferroptosis susceptibility in cellular populations. Mechanistically, TNF-α induces SLC7A11 and the glutamate-cysteine ligase modifier subunit by activating nuclear factor-κB (NF-κB), which upregulates the regulatory subunit of glutamate-cysteine ligase catalytic (GCLC) and glutamate-cysteine ligase catalytic, ultimately enhancing cellular GSH biosynthesis and protecting against lipid peroxidation-induced stress, thereby increasing ferroptosis resistance (89).

**Figure 6:**
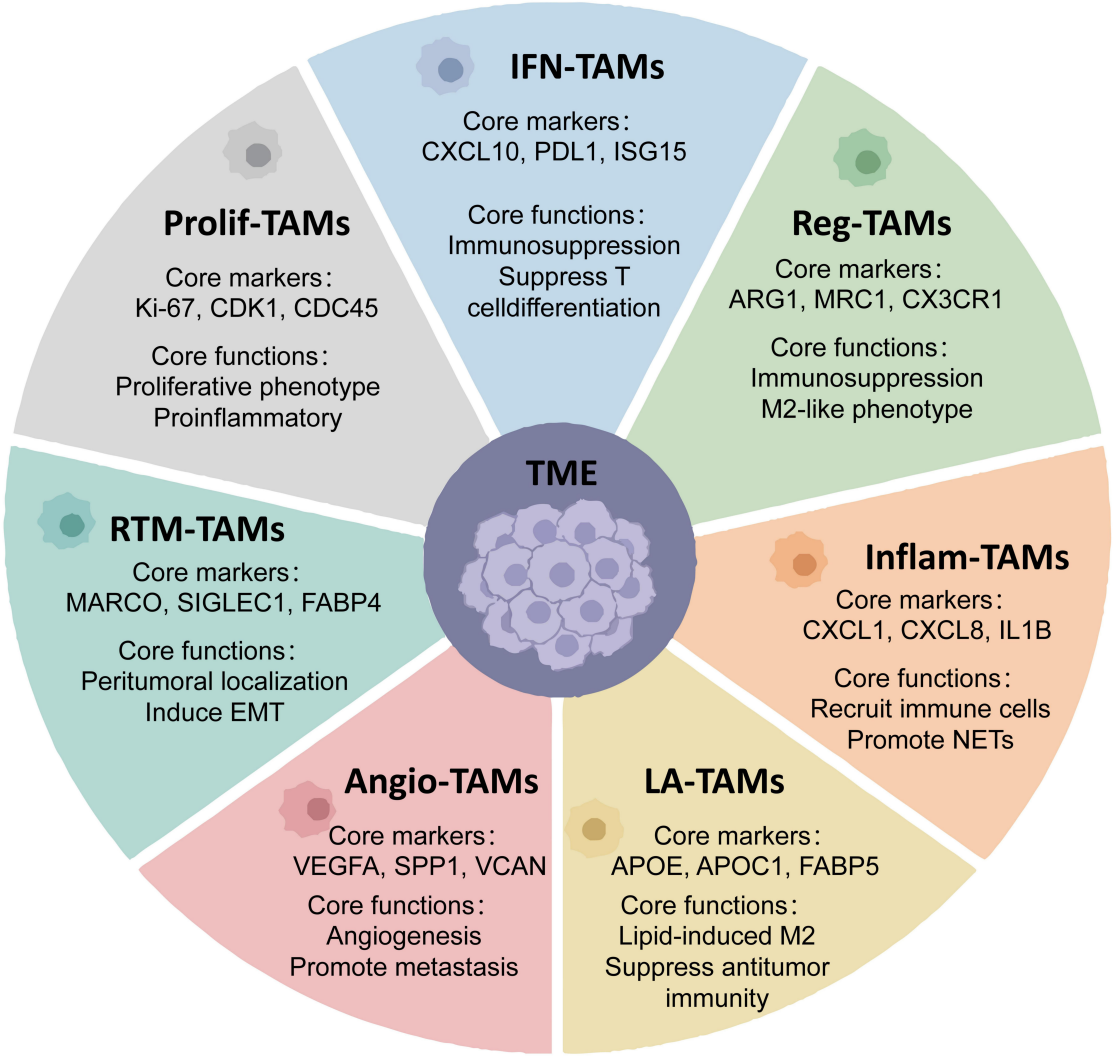
Tumor-associated macrophages (TAMs) exhibit diverse subtypes with distinct core markers and functions that modulate cancer development within the tumor microenvironment (TME). These include Reg TAMs, IFN-TAMs , Prolif-TAMs, Angio-TAMs, Inflam-TAMs, LA-TAMs, and RTM-TAMs. These macrophage phenotypes modulate tumor growth through various pathways.

In contrast, M2 macrophages, induced by IL-4, IL-13, IL-10, or glucocorticoids, produce anti-inflammatory cytokines, primarily TGF-β and IL-10 (90). By creating an immunosuppressive environment, M2 macrophages are frequently classified as tumor-associated macrophages (91). Their secreted TGF-β and IL-10 inhibit cytotoxic T lymphocytes and CD4+ T cells; however, some evidence suggests that TAMs include both M1 and M2 phenotypes (92). During tumor progression, macrophage-derived TGF-β1 can inhibit transcription through SMAD signaling, thereby promoting ferroptosis (85) and thus representing a complex interplay between immunosuppressive cytokines and ferroptotic mechanisms. M2 macrophages exhibit angiogenic and proinvasive properties, producing growth factors, chemokines, and MMPs that stimulate tumor growth, invasion, and metastasis (93), particularly facilitating tumor cell extravasation and growth in secondary sites. Different macrophage subpopulations regulate each process, and experimental studies demonstrating tumor inhibition through macrophage depletion confirm the critical role of tumor-immune cell interactions in cancer progression. Beyond the traditional M1-M2 paradigm, current transcriptomic analyses reveal greater TAM diversity with seven main subtypes: interferon-primed TAMs (IFN-TAMs), immune regulatory TAMs (Reg-TAMs), inflammatory cytokine-enriched TAMs (Inflam-TAMs), lipid-associated TAMs (LA-TAMs), proangiogenic TAMs (Angio-TAMs), RTM-like TAMs (RTM-TAMs), and proliferating TAMs (Prolif-TAMs) (**Figure 6**) (94).

This advanced classification provides a framework for understanding the diverse roles of macrophages in regulating ferroptotic. The phagocytic capacity of macrophages toward ferroptotic tumor cells demonstrates anti-tumor activity, with various immune-stimulatory signals released by ferroptotic tumor cells enhancing phagocytic efficiency (95). This ferroptosis-phagocytosis axis represents a promising anti-cancer therapeutic approach, potentially exploitable through the targeted induction of ferroptosis in tumor environments heavily infiltrated by specific macrophage subtypes.

## 6 Iron metabolism’s influence on macrophage polarization

Macrophages store iron through FT binding, with iron-related gene expression profiles varying across polarization stages. Compared to M2-type macrophages, M1-type macrophages exhibit elevated expression of hepcidin antimicrobial peptide (Hamp), ferritin heavy chain (FTH), and ferritin light chain (FTL), with reduced levels of FPN and iron regulatory proteins 1/2 (IRP1/2), demonstrating enhanced iron storage capacity (96). Iron overload induces M1 polarization, as confirmed by studies showing increased M1 markers, such as IL-6, TNF-α, and IL-1β, with a concurrent reduction in M2 markers, including tissue transglutaminase 2 (TGM2), effectively promoting M1-type macrophage polarization (97). Iron overload triggers the production of inflammatory factors and encourages the development of an M1 phenotype by stimulating glycolysis, thereby accelerating the progression of atherosclerosis. Iron overload-induced ROS generation, similar to p53 acetylation, also promotes M1 polarization (98, 99). However, iron overload doesn’t invariably lead to M1-type polarization; some studies indicate that under chronic iron overload conditions, THP-1 monocyte-derived macrophages often display M2-type characteristics with downregulated M1-type macrophage markers (100).

### 6.1 Macrophage recruitment mechanisms initiated by ferroptotic cells

Ferroptosis occurs in various disease states, with macrophages responsible for ferroptotic cell clearance. Ferroptotic cells activate macrophage functions and recruitment through damage-associated molecular patterns (DAMPs), which are endogenous danger signals that recruit and activate macrophages, thereby initiating immune defense mechanisms. Studies demonstrate that during ferroptosis, ferroptotic cells release the autophagy-dependent DAMP high-mobility group box 1 (HMGB1). HMGB1-mediated macrophage inflammation requires the receptor for advanced glycation end products (101, 102). In ferroptosis response, macrophage Toll-like receptor 2 (TLR2) initially interacts with oxidized phospholipid peroxides and 1-stearoyl-2-15-hPEtE-sn-glycero-3-phosphatidyl-ethanolamine (SAPE-OOH) on ferroptosis cell surfaces, enhancing macrophage phagocytosis efficiency. Notably, the depletion of anti-HMGB1 neutralizing antibody or arginine catabolism enzyme (AGRE) mitigates macrophage inflammatory responses, suggesting that restricting HMGB1 expression may be a potential approach for managing macrophage inflammation (103, 104).

Beyond HMGB1, ferroptosis cells trigger inflammatory responses and promote macrophage recruitment by activating molecular pathways that induce inflammation. They induce the expression of inflammation-related genes, including CCL2 and CCL7, which enhance macrophage recruitment and chemotaxis (105).

## 7 Ferroptosis’s bidirectional relationship with macrophage function

### 7.1 Iron homeostasis controls macrophage polarization

Iron metabolism is quite crucial for macrophage phenotypic differentiation. Essential micronutrient iron coordinates several cellular functions, including proliferation, metabolic activity, and cellular differentiation. The physiological iron supply mostly depends on macrophage-mediated recycling of erythrocyte iron over complex regulatory routes. Especially in embryonic and differentiation settings, iron availability significantly affects the determination of macrophage destiny and functional programming. Macrophages first store iron intracellularly, mostly in ferritin (Ft) complexes. Iron-regulating gene expression profiles exhibit distinct trends corresponding to the stages of macrophage polarization. With concurrent downregulation of ferroportin (FPN) and iron-regulating proteins (IRP1/2), M1-polarized macrophages show increased hepcidin (Hamp) and ferritin heavy/light chains (FTH/FTL) relative to their M2 counterparts (96).

Usually, iron abundance favors M1 polarization. Excessive iron loading increases the expression of M1-associated inflammatory mediators, including IL-6, TNF-α, and IL-1β, while suppressing M2 markers such as transglutaminase 2 (TGM2), so efficiently guiding polarization toward the pro-inflammatory M1 phenotype (97). Apart from inflammatory cytokine production, iron excess has been shown to exacerbate atherosclerosis development by enhancing glycolytic metabolism, thereby supporting the M1 phenotype (98). Furthermore, iron overload promotes M1 polarization using enhanced p53 acetylation and reactive oxygen species production (99). However, the link between iron loading and macrophage polarization exhibits context dependence; studies from 2020 demonstrate that chronic iron excess conditions induce an M2-like phenotype in THP-1 monocyte-derived macrophages, concurrently downregulating M1 markers (100).

### 7.2 Ferroptotic cells program macrophage mobilization

**Figure 7:**
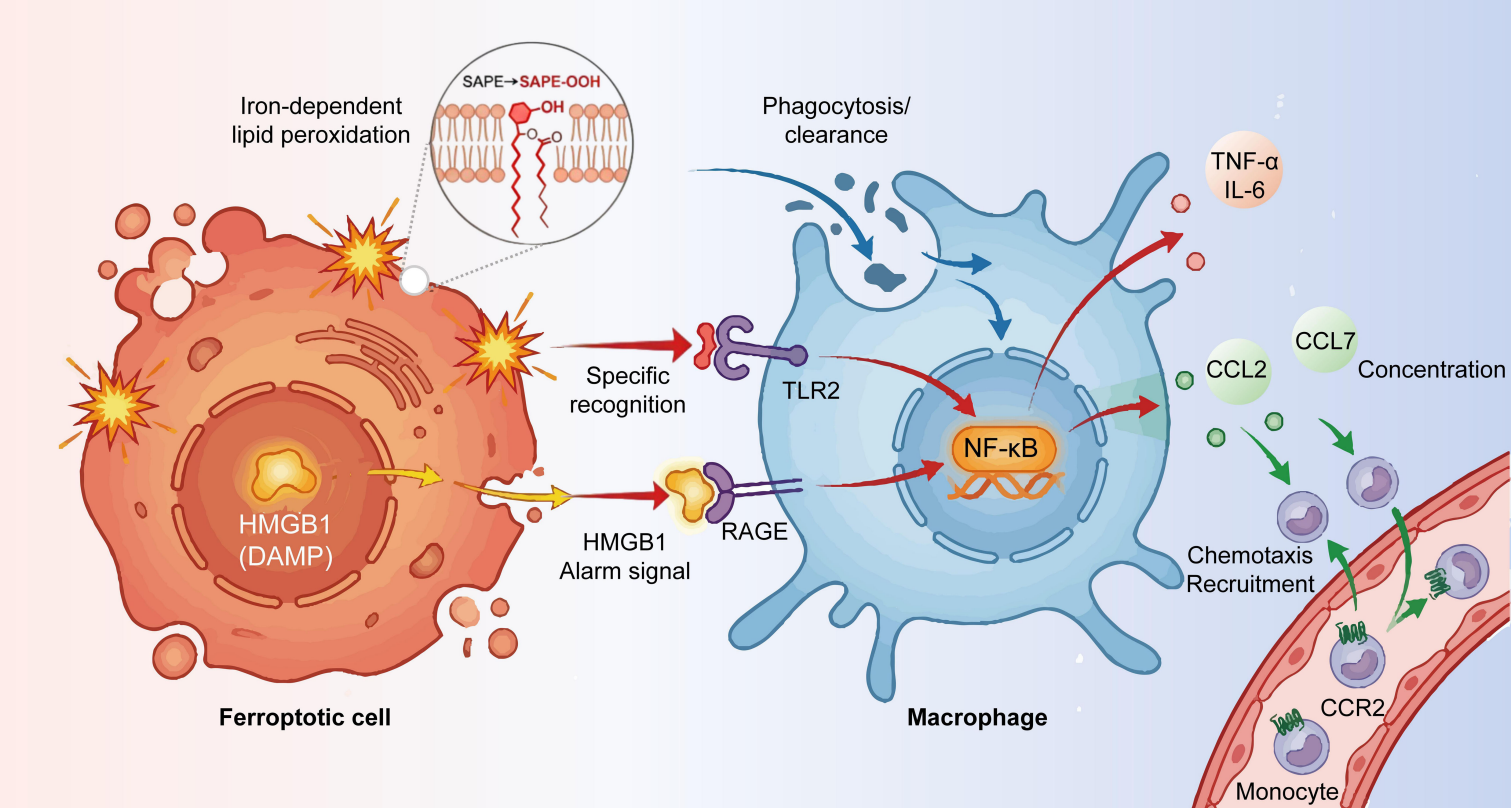
Ferroptotic cells (with SAPE-OOH-mediated lipid peroxidation) release HMGB1, which binds macrophages’ TLR2/RAGE to promote engulfment and trigger NF-κB-dependent inflammatory responses (cytokine secretion) plus monocyte recruitment via chemokines.

Many clinical diseases cause ferroptotic cell death, and macrophages are the primary effectors in ferroptotic cell clearance. As previously mentioned, the macrophage’s phagocytic ability is a cornerstone of immunological surveillance. Ferroptotic cells actively influence macrophage functional responses and recruitment dynamics (**Figure 7**). As endogenous danger signals, damage-associated molecular pattern molecules (DAMPs) enable macrophage recruitment and activation, alerting the immune system. During ferroptosis, cells release the DAMP high-mobility group box 1 (HMGB1) by autophagy-dependent processes. The sophisticated glycosylation end-product-specific receptor controls macrophage inflammatory responses by HMGB1 (101).

Present on ferroptotic cell surfaces, macrophage toll-like receptor 2 (TLR2) initially interacts with oxidized phospholipids, specifically 1-stearoyl-2-15-HpETE-sn-glycero-3-phosphatidyl-ethanolamine (SAPE-OOH), therefore improving phagocytic effectiveness. Moreover, neutralizing HMGB1 with specific antibodies or depleting HMGB1 reduces inflammatory activation in macrophages, implying that HMGB1 inhibition may be a possible therapeutic strategy for controlling macrophage-mediated inflammation (103). Beyond HMGB1 signaling, ferroptotic cells activate other molecular inflammatory pathways, triggering inflammatory reactions and the recruitment of macrophages. With a special focus on chemokines CCL2 and CCL7, which facilitate macrophage chemotaxis and recruitment, ferroptosis enhances the expression of many inflammation-associated genes (102).

### 7.3 Targeting macrophage metabolism modulates ferroptosis in cancer

The metabolic diversity of TAM subsets provides multiple therapeutic entry points for modulating ferroptosis in cancer cells. M1-like macrophages, characterized by elevated glycolysis, disrupted TCA cycle intermediates, enhanced ferritinophagy, and high ROS/NO production, naturally promote ferroptotic pressure. Therapeutically, these cells can be further potentiated using agents that intensify iron release (e.g., NCOA4 activators), enhance ACSL4-mediated PUFA incorporation, or inhibit GPX4-stabilizing pathways (106). Targeting metabolic checkpoints such as PKM2 or succinate oxidation can increase the availability of redox-active metabolites that accelerate lipid peroxidation in tumor cells. In contrast, M2-like macrophages, driven by oxidative phosphorylation, fatty-acid β-oxidation, and glutathione synthesis, constitute a ferroptosis-inhibitory niche. These cells can be reprogrammed metabolically using FAO inhibitors (etomoxir), glutathione-depleting drugs (buthionine sulfoximine), or ferroportin blockers to decrease iron efflux. Suppression of IL-10/STAT3 or TGF-β/SMAD-dependent antioxidant circuits enables the restoration of lipid peroxidation susceptibility in adjacent tumor cells (107). Additionally, M2-associated iron recycling pathways (HMOX1 upregulation, heme metabolism) can be targeted to shift intracellular iron pools toward pro-ferroptotic states. Lipid-associated TAMs (LA-TAMs) represent a third emerging subset with high expression of lipid uptake receptors, cholesterol efflux transporters, and PPAR-driven immunosuppressive programs (108). Therapeutic interventions aimed at modulating lipid availability, such as ACAT inhibitors, PPAR antagonists, or scavenger-receptor blockade, can reduce the supply of anti-ferroptotic lipid mediators and promote oxidative lipid stress within tumor cells.

Collectively, these strategies demonstrate that metabolically targeting TAM subsets is a powerful approach to controlling ferroptosis, suggesting multiple avenues for combination therapies that integrate ferroptosis inducers, immune checkpoint modulators, and metabolic inhibitors.

## 8 Therapeutic approaches targeting macrophage-ferroptosis interactions

Macrophages enhance the efficacy of anti-tumor treatments by remodeling the tumor microenvironment. For instance, targeted nanoparticles enable the expression of cancer cell surface receptors by adding specific ligands. Radiotherapy increases tumor cell ferroptosis susceptibility, significantly inducing the expression of long-chain acyl-CoA synthetase 4 (ACSL4) and the ferroptosis inhibitors SLC7A11 and GPX4, thereby effectively suppressing tumor growth (109). Studies show that immune checkpoint blockade therapy activates CD8+ T cells to secrete IFN-γ, promoting ACSL4 growth, regulating cellular acyl-chain phospholipid-fatty acid binding to arachidonic acid (AA), and inducing tumor cell ferroptosis (110). Combined macrophage and ferroptosis-targeted therapy effectively treats tumors. Combination drug therapy is commonly used to address various cancers and infectious diseases (111). For example, they designed m@Au-D/B nanoparticles that induce effective ferroptosis and immune responses by incorporating L-buthionine-(S, R)-sulfoximine (BSO) and doxorubicin (DOX). By triggering ferroptosis through GSH depletion and ROS accumulation, photothermal therapy, combined with ROS repolarization, converts TAMs from M2 to an M1 phenotype (112). These targeted nanoparticles initiate combined cancer therapy through ferroptosis and TAM repolarization mechanisms, providing an advantageous anti-cancer approach.

Researchers successfully repolarized macrophages from tumor-promoting M2 to anti-tumor M1 phenotype through ferroptosis using MIL88B/RSL3 nanomaterials. This ferroptosis-enhanced macrophage regulation strategy may apply to other iron-based nanomaterials and iron-related lethal agent combinations. Recent research has identified CD24-overexpressing cells as being resistant to paclitaxel but sensitive to ferroptosis agonists. A precision-targeted therapy system targeting CD24 was designed to enhance cell ferroptosis and macrophage phagocytosis through the NF2-YAP signaling axis by inhibiting FSP1 and CD24, ultimately leading to cell death, inhibiting TNBC tumor growth, and potentially eliminating certain tumors (113). Recent studies demonstrate that ROS-responsive micelles simultaneously load the sonosensitizing agent protoporphyrin IX (PPIX) as an initiating drug, utilizing macrophages as active targeting vectors for delivering the treatment to the site of rheumatoid arthritis (RA). Macrophage co-incubation prepared the PEG-PPS-Fe_3_O_4_-PPIX@M drug delivery system for synergistic RA treatment combining sonodynamic therapy and ferroptosis (114).

### 8.1 Ferroptosis in cancer treatment

**Figure 8:**
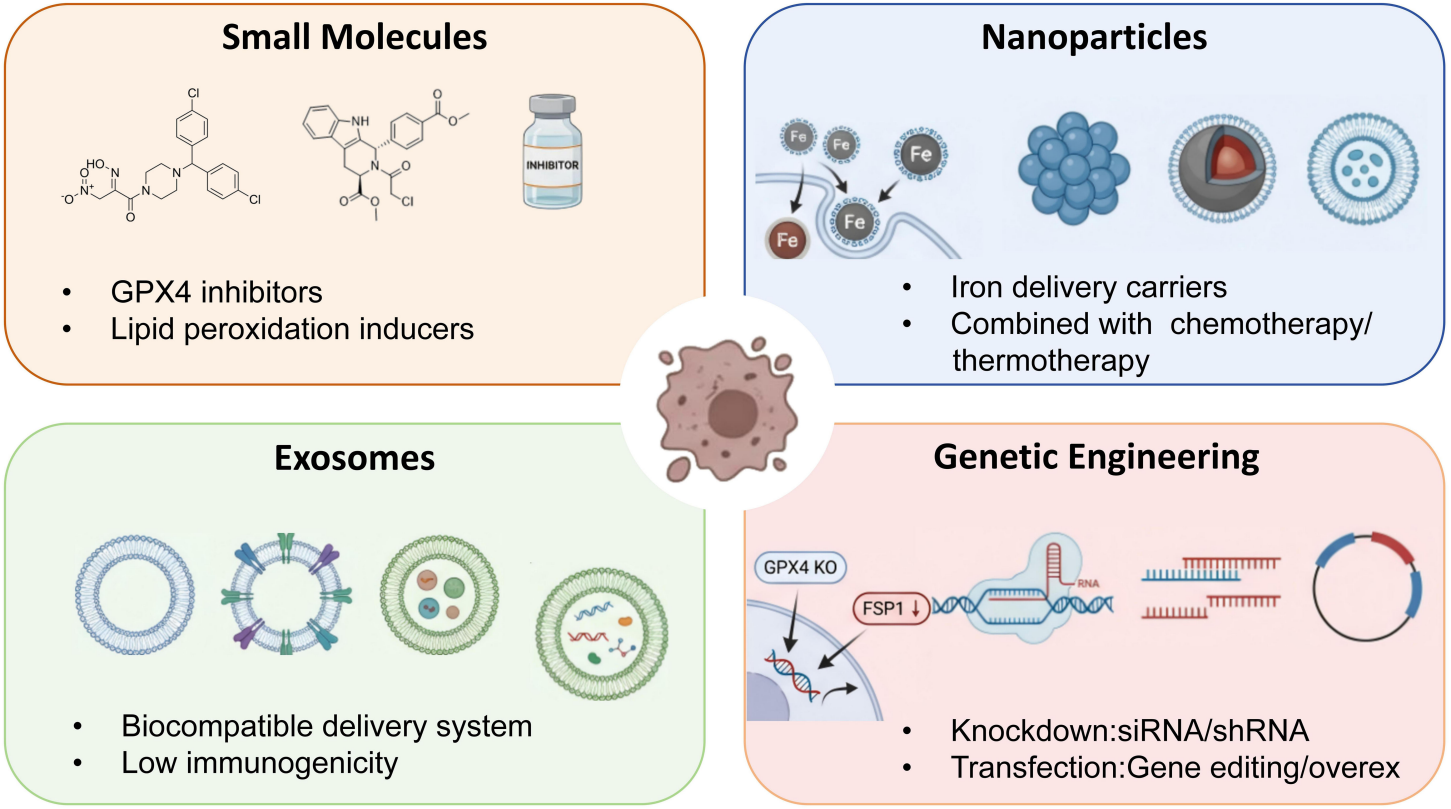
The potential application of ferroptosis in oncological therapy. A depiction of ferroptosis-oriented cancer therapies, encompassing small molecules, nanoparticles, exosomes, and genetic engineering. Nanomaterials can be utilized as agents to induce ferroptosis and as iron transporters in chemotherapy, in conjunction with heat and autophagy. Exosomes exhibit superior biocompatibility and reduced immunogenicity compared to nanomaterials, enhancing their potential for clinical trial applications. One of the most challenging aspects of gene technology is its division into two categories: “knockdown” and “transfection.” It will encounter a range of challenges.

The maintenance of cellular homeostasis and the prevention of proliferative diseases fundamentally rely on the controlled process of cell death (115). Cancer cells undergo a variety of regulated cell death processes during development, such as necrosis and apoptosis. Utilizing specific CCD pathways is a powerful and long-lasting approach to treating cancer. Although some contemporary anti-cancer drugs focus on apoptotic signals (116), newer studies emphasize that generating Ferroptosis is a possible innovative anti-cancer strategy, thus creating new therapeutic possibilities (117). Ferroptosis induced by tiny chemical substances, nanomaterials, exosomes, and genetic technology has shown considerable anti-tumor efficacy (**Figure 8**).

### 8.2 Nanoparticle-mediated ferroptosis

Nanoparticles (NPs) exhibit outstanding efficacy in precise targeting through both active and passive mechanisms. Although nanoparticle drugs have been rapidly incorporated into cancer treatment, they have encountered obstacles, including immunogenicity and cytotoxicity (118). The complexity of tumors requires the combination of Ferroptosis with other treatments. An innovative approach that combines Ferroptosis with photothermal therapy (PTT) using SRF@MPDA-SPIO-NPs, SPIO promotes Ferroptosis, and SRF induces Ferroptosis (119). MPDA-NPs provide adaptive photothermal therapy through laser-induced heat generation. The PTT-ferroptosis combination shows considerable anti-tumor potential. The activation of autophagy can also accelerate ferritin apoptosis by promoting the degradation of ferritin. Nanocomposites MnO2@HMCu2-xS (HMCM) have been used in cancer treatment by photothermal therapy (PTT) and autophagy-enhanced ferroapoptosis. MnO2 induces ferroapoptosis in the tumor microenvironment (TME) by consuming GSH, while photothermal therapy (PTT) and HMCU2-XS-induced ferroapoptosis have a synergistic effect. Ferroptosis also generates reactive oxygen species (ROS) by releasing Mn2+, serving as a supplementary mechanism for Ferroptosis induced by lipid hydrogen peroxide (120). In cancer treatment, genetic technologies include gene knockout and gene transfection methods (121). The primary genetic targets for treating ferroptosis may include p53, Gpx4, ACSL4, and Nrf2.

### 8.3 Exosomes in ferroptosis

Regulation. Although nanotechnology has enhanced cancer treatment, the use of nanomaterials in this technology may exhibit cytotoxicity (122). Exosomes (30–120 nm lipid bilayer vesicles) possess superior biocompatibility, low immunogenicity, and tumor-targeting ability, making them the best drug delivery carriers. Traditional hormone and EGFR-targeted therapies are usually ineffective for triple-negative breast cancer (TNBC). A method named rastin@FA-Exo can enhance exosome transport and folate receptor uptake in triple-negative breast cancer (TNBC) cells, thereby enabling targeted drug delivery. Conversely, exosomes may also confer resistance to ferroptosis in tumor cells (123). Ferritin is an iron storage protein that was detected in exosomes of anti-apoptosis cells treated with Gpx inhibitors. During the induction of ptosis, the level of protruding protein 2 was negatively correlated with cellular iron. Cells continuously overexpress the iron export mechanism by transporting ferritin and iron through MVBs/exosomes, thereby limiting the accumulation of iron within the cells and inhibiting ferroptosis. Inactivating this pathway, whether spontaneously or through intervention, triggers factors that induce ferroptosis, underscoring the importance of regulating ferroptosis in cancer treatment (124). Under the stimulation of ferroptosis, the expression of protruding protein-2 in breast cancer cells increases, which can enhance the production of MVB/exosomes containing ferritin and promote iron excretion (125). The ferritin cascade, mediated by MVB-exosomes and Protrusion protein-2, may represent a novel approach to suppressing cancer by inhibiting Ferroptosis.

### 8.4 Small molecules inducing ferroptosis

Resistance to radiotherapy and chemotherapy can lead to treatment failure, making Ferroptosis a new target for traditional treatment (126). Sorafenib is the primary therapeutic agent for advanced hepatocellular carcinoma (HCC), which can induce cell apoptosis, inhibit cell growth, and trigger ferroptosis. Nrf2, MTIG, and Rb have been proven to alleviate sorafenib-induced Ferroptosis. Inhibiting these regulatory factors may increase resistance to sorafenib. SAS inhibits xCT and is used for the treatment of arthritis and inflammatory bowel disease, which can lead to ferroptosis, a potential cancer treatment method, as shown in **Table 2**. Inhibition of CISD2 in HNCC can increase mitochondrial Fe2+ and reactive oxygen species (ROS), thereby enhancing the sensitivity of malignant tumors to Ferroptosis induced by SAS (127). Artemisinin and its derivatives can induce Ferroptosis by increasing reactive oxygen species.

**Table 2:** Current and emerging therapeutic strategies targeting TAM-ferroptosis axis.

Strategy category	Specific approach	Mechanism	Current status	Key examples	Clinical outcomes
**Nanoparticle-Mediated**	Iron oxide nanoparticles	M2→M1 repolarization + ferroptosis	Clinical trials	Ferumoxytol, SRF@MPDA-SPIO-NPs	Tumor growth inhibition
	MIL88B/RSL3 nanomaterials	Enhanced ferroptosis + TAM regulation	Preclinical	MnO2@HMCu2-xS (HMCM)	Synergistic anti-tumor effect
**Genetic Modifications**	GPX4 knockdown	Direct ferroptosis induction	Research phase	CRISPR/siRNA approaches	Enhanced tumor sensitivity
	p53/ACSL4/NRF2 modulation	Ferroptosis pathway regulation	Preclinical	Gene therapy vectors	Tumor-specific targeting
**Exosome-Based**	Rastin@FA-Exo	Targeted drug delivery to TNBC	Preclinical	Folate receptor targeting	Improved biocompatibility
	Anti-ferritin exosome therapy	Block iron export resistance	Research phase	Neutralizing ferritin transport	Overcome drug resistance
**Small Molecules**	Sorafenib	Multi-target: apoptosis + ferroptosis	FDA approved	HCC first-line therapy	Extended survival
	Sulfasalazine (SAS)	xCT inhibition	FDA approved (IBD)	Repurposed for cancer	Phase II trials
Artemisinin derivatives	ROS-mediated ferroptosis	Clinical trials	Natural product derivatives	Combination therapy potential
**Combination Therapies**	Radiotherapy + ferroptosis	Enhanced ACSL4 expression	Clinical practice	RT + sorafenib	Synergistic efficacy
	ICB + ferroptosis inducers	IFN-γ-mediated enhancement	Clinical trials	Anti-PD1 + erastin analogs	Improved response rates
**TAM Repolarization**	m@Au-D/B nanoparticles	Photothermal + M2→M1 switch	Preclinical	BSO + DOX combination	Dual anti-tumor mechanism

## 9 The enigmatic interplay: macrophages and ferroptosis in disease

**Figure 9:**
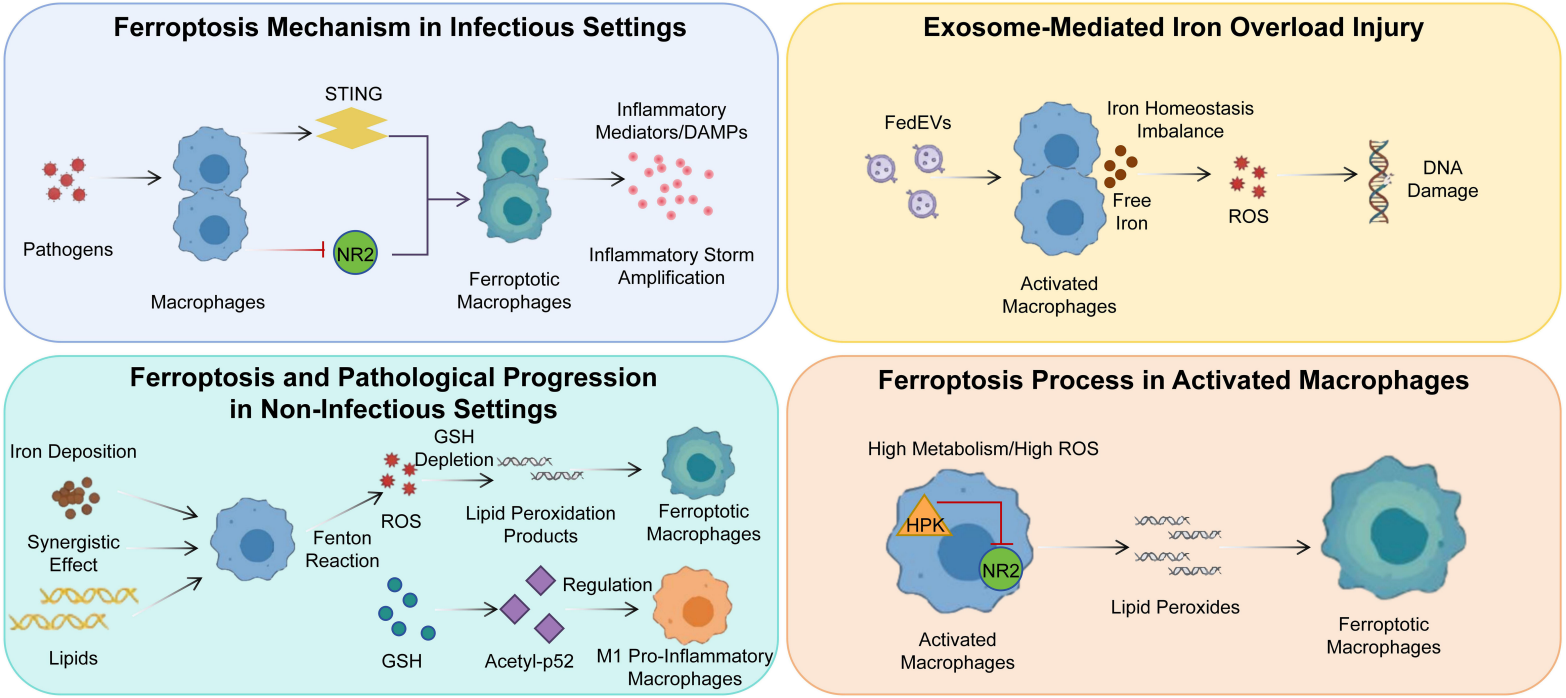
The diverse roles and mechanisms of macrophage ferroptosis in cancer disease.

Ferroptosis, a distinct form of regulated cell death, holds significant importance in the pathology of various diseases, particularly in oncology, as illustrated in **Figure 9**. This has spurred considerable interest in devising novel therapeutic interventions that modulate its intricate regulatory pathways. However, the precise control of ferroptosis, including its induction, inhibition, and fine-tuning, remains an area of ongoing scientific discourse (128).

While the impact of ferroptosis on malignant cells has been extensively explored, the reciprocal relationship between ferroptosis and macrophages, critical immune cells that profoundly influence disease trajectory and prognosis, has received comparatively less attention. This oversight underscores a crucial knowledge gap.

The mechanisms governing macrophage-mediated phagocytosis of ferroptotic cells are still nascent and incomplete. Currently, sulfated phosphatidyl-ethanolamine hydroperoxide (SAPE-OOH) on the surface of ferroptotic cells is recognized as an “eat-me” signal, facilitating interaction with Toll-like receptor 2 (TLR2) on macrophages (129). Nevertheless, the potential involvement of other classical phagocytic cues, such as oxidized phosphatidylserines (oxPSs), warrants further rigorous investigation. Furthermore, while the presence of “do not eat me” signals, such as CD47 and PD-L1, is known to regulate phagocytosis in other cellular contexts, their existence and role in ferroptotic cells remain largely uncharacterized. Elucidating these inhibitory signals is vital for a comprehensive understanding of the crosstalk between ferroptosis and macrophages. Beyond cellular recognition, the complex interplay among ferroptosis regulatory mechanisms, the immunosuppressive tumor microenvironment, and resistance to immunotherapy represents another critical frontier requiring more profound elucidation. A nuanced understanding of these interconnected processes is imperative for advancing therapeutic strategies across diverse disease landscapes.

## 10 Conclusion and future perspectives

The immunometabolic crosstalk between tumor-associated macrophages (TAMs) and ferroptotic cancer cells has emerged as a defining regulator of tumor fate, influencing progression, metastasis, immune evasion, and therapeutic response. TAMs shape ferroptotic sensitivity by controlling iron transport, redox buffering, glutathione metabolism, lipid peroxidation pathways, and mitochondrial stress signaling. In turn, ferroptotic cancer cells release oxidized phospholipids, iron-rich vesicles, and damage-associated molecular patterns that remodel macrophage polarization and inflammatory tone. This reciprocal communication integrates metabolic reprogramming with immune regulation, positioning ferroptosis as a key determinant of macrophage-driven tumor biology. Despite significant advances, essential gaps remain unresolved. Current knowledge only partially explains how distinct TAM subsets, such as inflammatory M1-like macrophages, immunosuppressive M2-like macrophages, lipid-associated TAMs, and embryonically derived tissue-resident macrophages, differ in their ability to induce or restrain ferroptosis within tumors. Moreover, the spatial and temporal dynamics of TAM-ferroptosis interactions during tumor initiation, metastatic dissemination, treatment resistance, or immunotherapy response remain poorly characterized. Continued progress will depend on technologies such as single-cell multi-omics, spatial proteogenomics, intravital imaging of iron flux, and ferroptosis-reporter mouse models, which can resolve these interactions with high precision.

Looking forward, therapeutic opportunities are expanding rapidly. Targeting metabolic vulnerabilities in specific macrophage subsets, such as limiting ferroportin-mediated iron export in M2 TAMs or enhancing ACSL4-dependent lipid peroxidation in inflammatory TAMs, may shift the TME toward a ferroptosis-permissive state. Nanomedicine platforms that co-deliver ferroptosis inducers with TAM-repolarization agents, as well as rational combinations with immune checkpoint inhibitors, radiotherapy, or cytokine modulators, show strong potential for durable anti-tumor responses. Equally important is the development of biomarkers reflecting iron-handling capacity, lipid oxidative signatures, or ferroptosis-associated cytokine patterns to guide patient stratification and optimize therapy.

Collectively, advancing the mechanistic understanding and therapeutic exploitation of the TAM-ferroptosis axis holds considerable promise for the next generation of precision immunometabolic cancer treatments.
